# Supplementary material for: Phasing of de novo mutations using a scaled‐up multiple amplicon long‐read sequencing approach
Source: Hum Mutat. 2022 Sep 14;43(11):1545–56. doi: 10.1002/humu.24450 (PMC9826063; doi:10.1002/humu.24450)
Supplement: Supplementary file 1 — Supplementary information. [file HUMU-43-1545-s002.pdf]

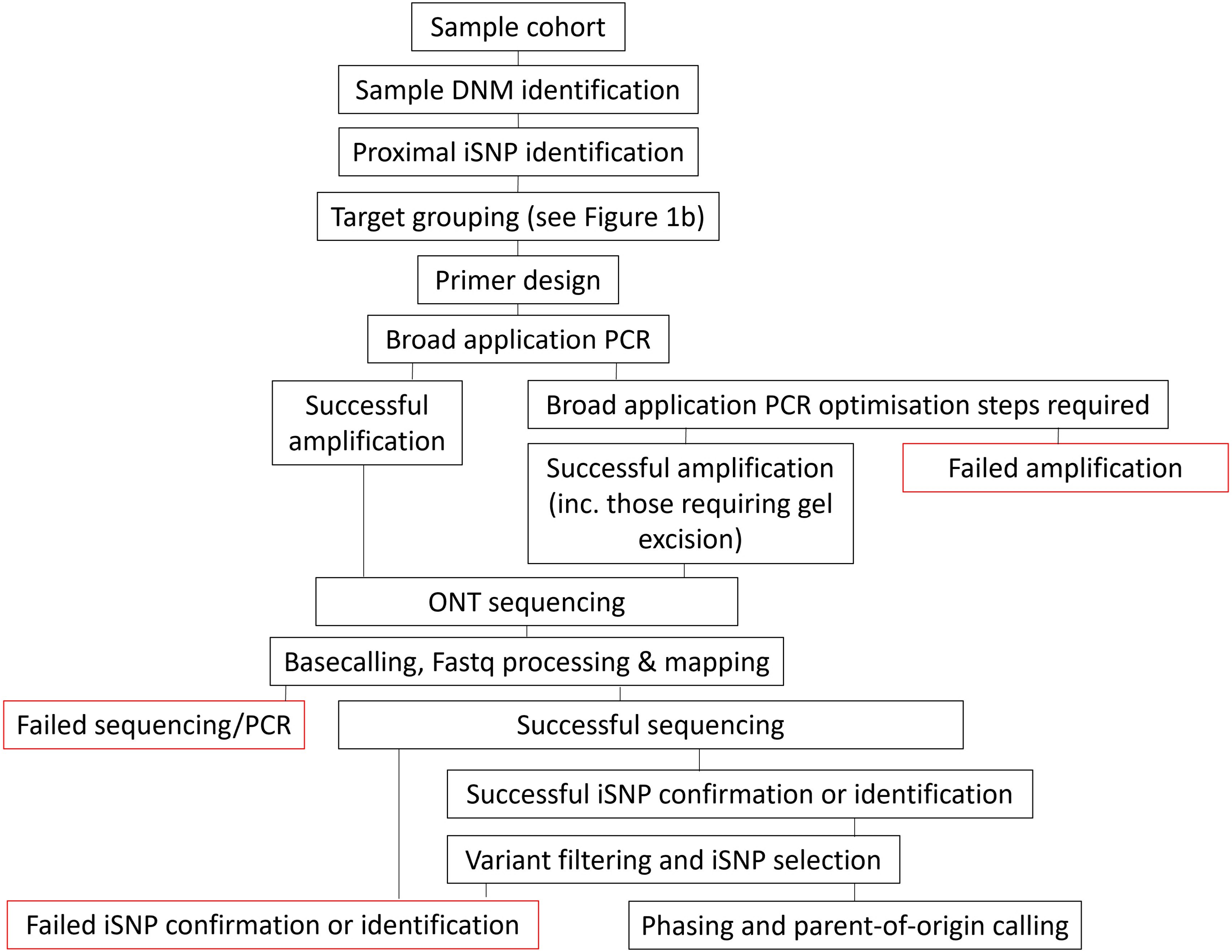

Supplementary Figure 1 **Basic overview of the long-read targeted phasing approach**. Red boxes highlighting failed sample outcomes.

(a)

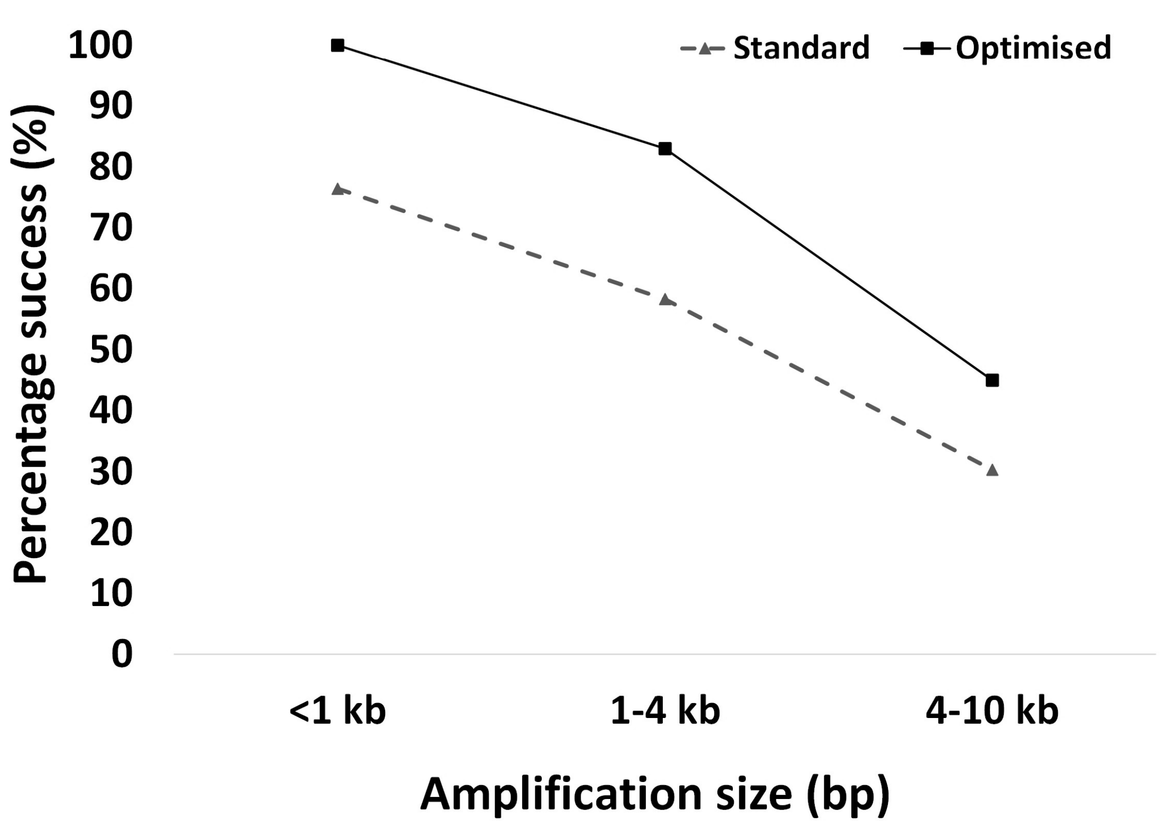

(b)

PCR optimisation steps	Optimisation step success (%)	Total PCR success (%)
Long-range PCR	50	50
Dilution (1ng/µl)	50	75
Dilution range (2, 4, and 6 ng/µl)	30	83
Hot start polymerase	32	88
Primer pair specific optimisation	62	95

5% require new primer design

Supplementary Figure 2 **Long range PCR performance and optimisation. (a)** change standard to rapid. Percentage comparison of first-time primer success using our standardised approach and primer success after optimisations at increasing target sizes (kb). **(b)** Table of optimisation categories, displaying total target amplification success rates at each stage.

(a)

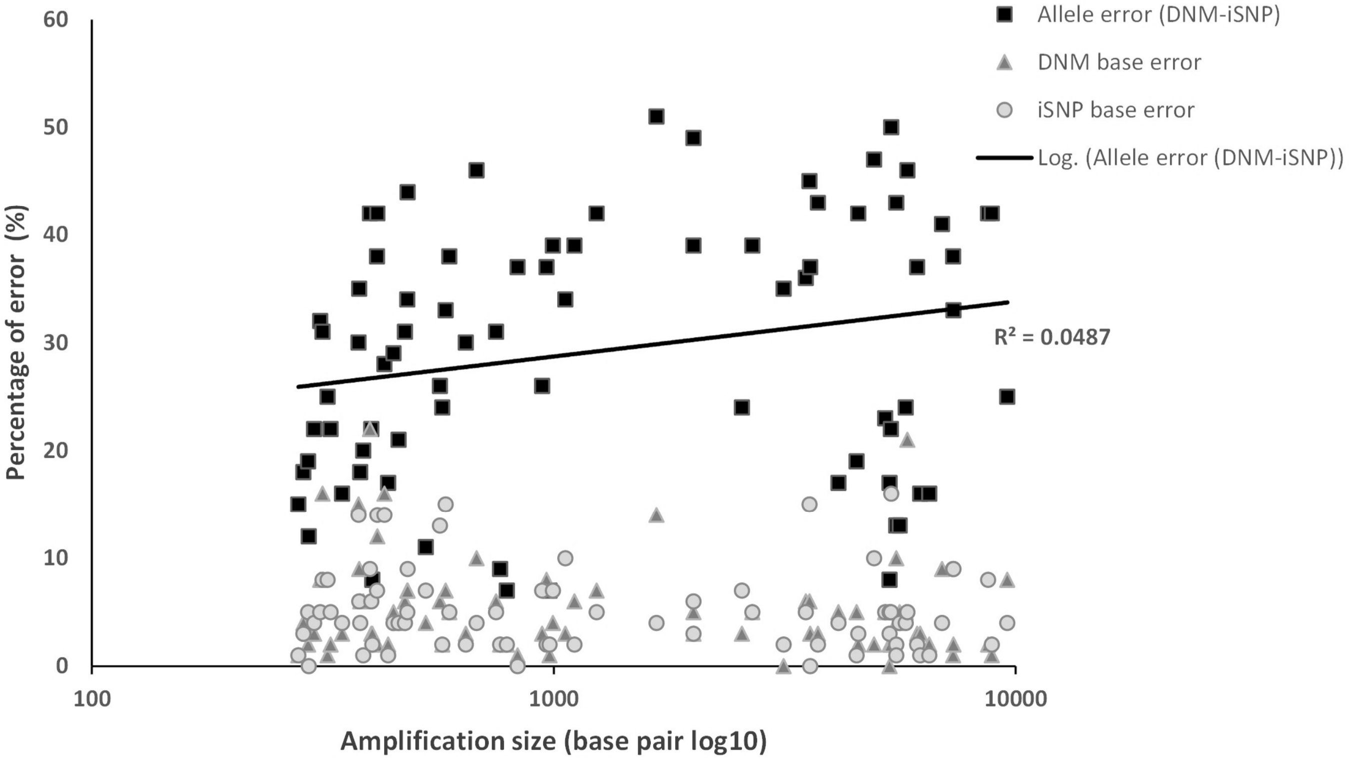

(b)

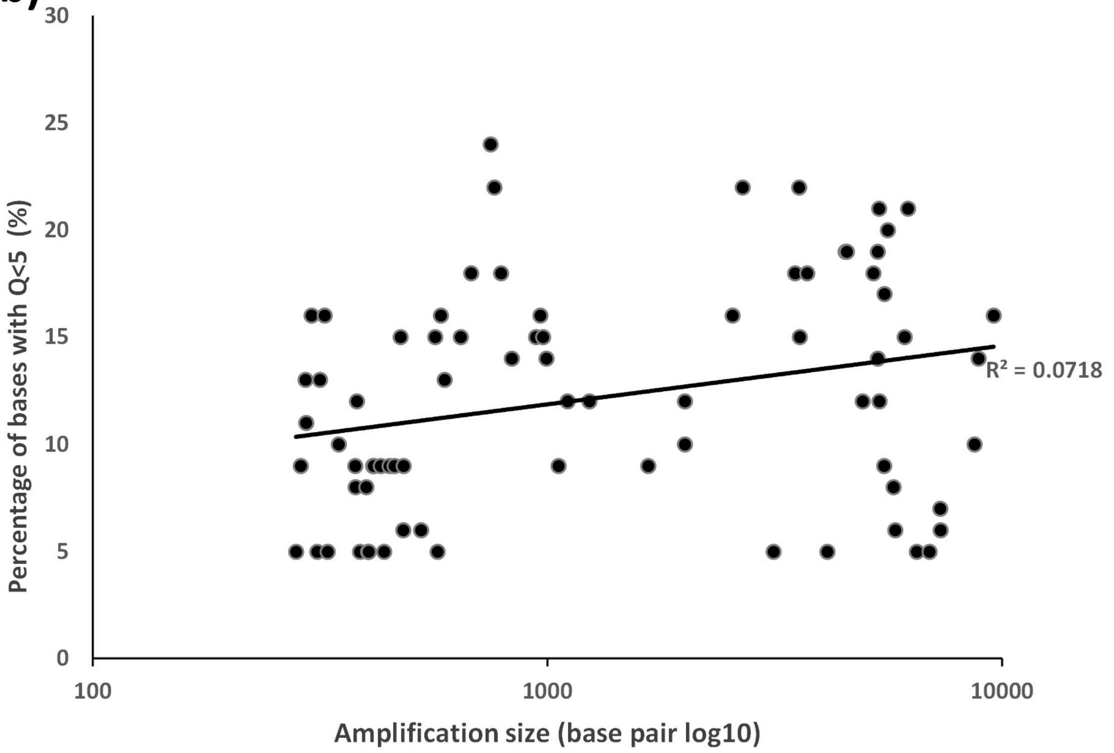

Supplementary Figure 3 **a)** Log10 scatter plot of target amplification sizes in relation to the percentage of false allele coverage of each target and the percentage of false iSNP/DNM base coverage of each target. The trend of the data is highlighted by the logarithmic line of best fit. **b)** Log10 scatter plot of target amplification sizes in relation to the percentage of bases with quality score <5 (see method section 'Bioinformatics'). The trend of the data is highlighted by the logarithmic line of best fit.

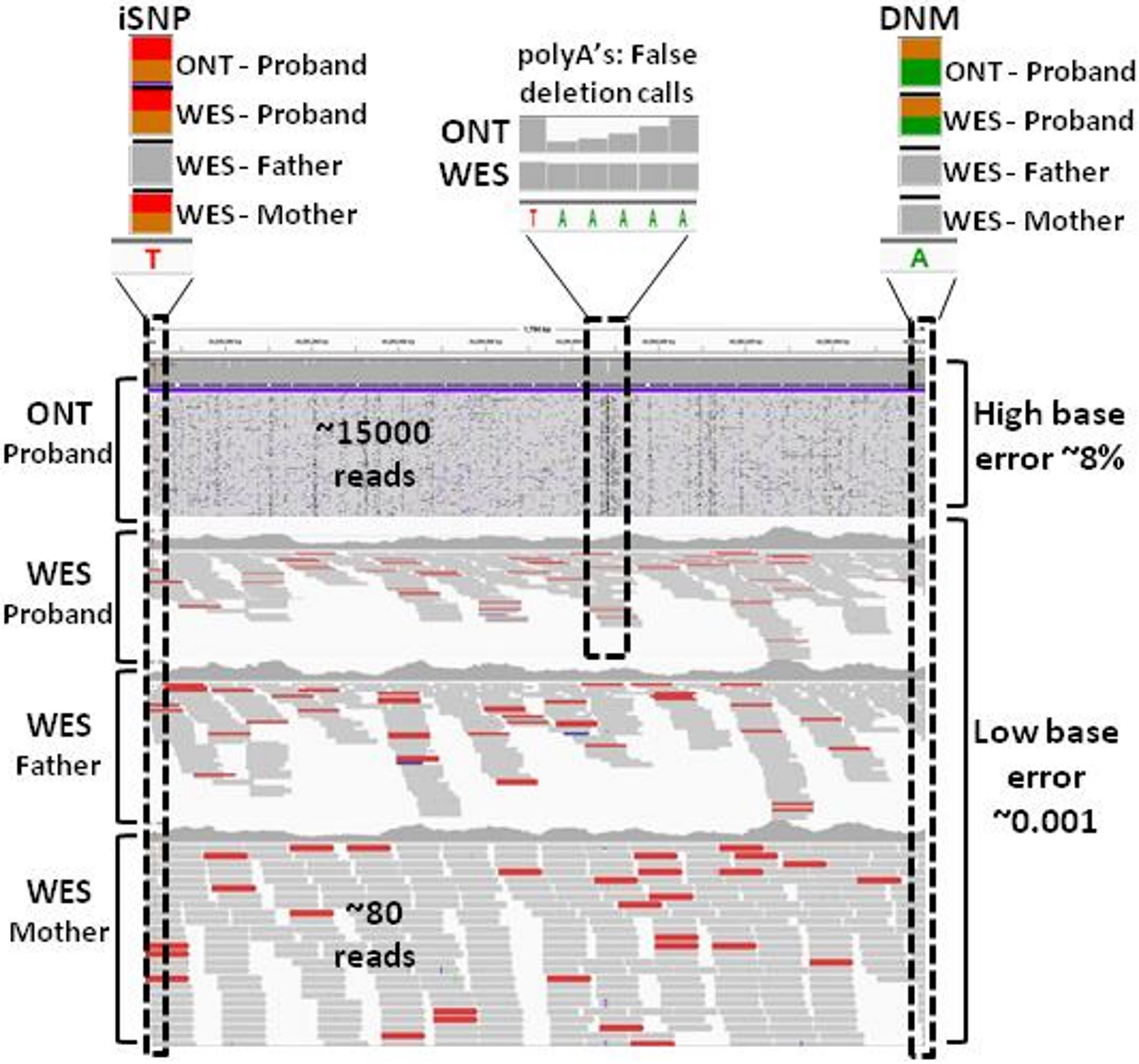

Supplementary Figure 4 **IGV illustration of a DNM showing a comparison of WES and ONT data.** Using long-read targeted sequencing, the ~1700 bp region that these variants span was sequenced and visualized in IGV. Approximately 15000 reads are phased in the ONT data, and the quality and coverage differences between those reads and the WES reads can be observed.

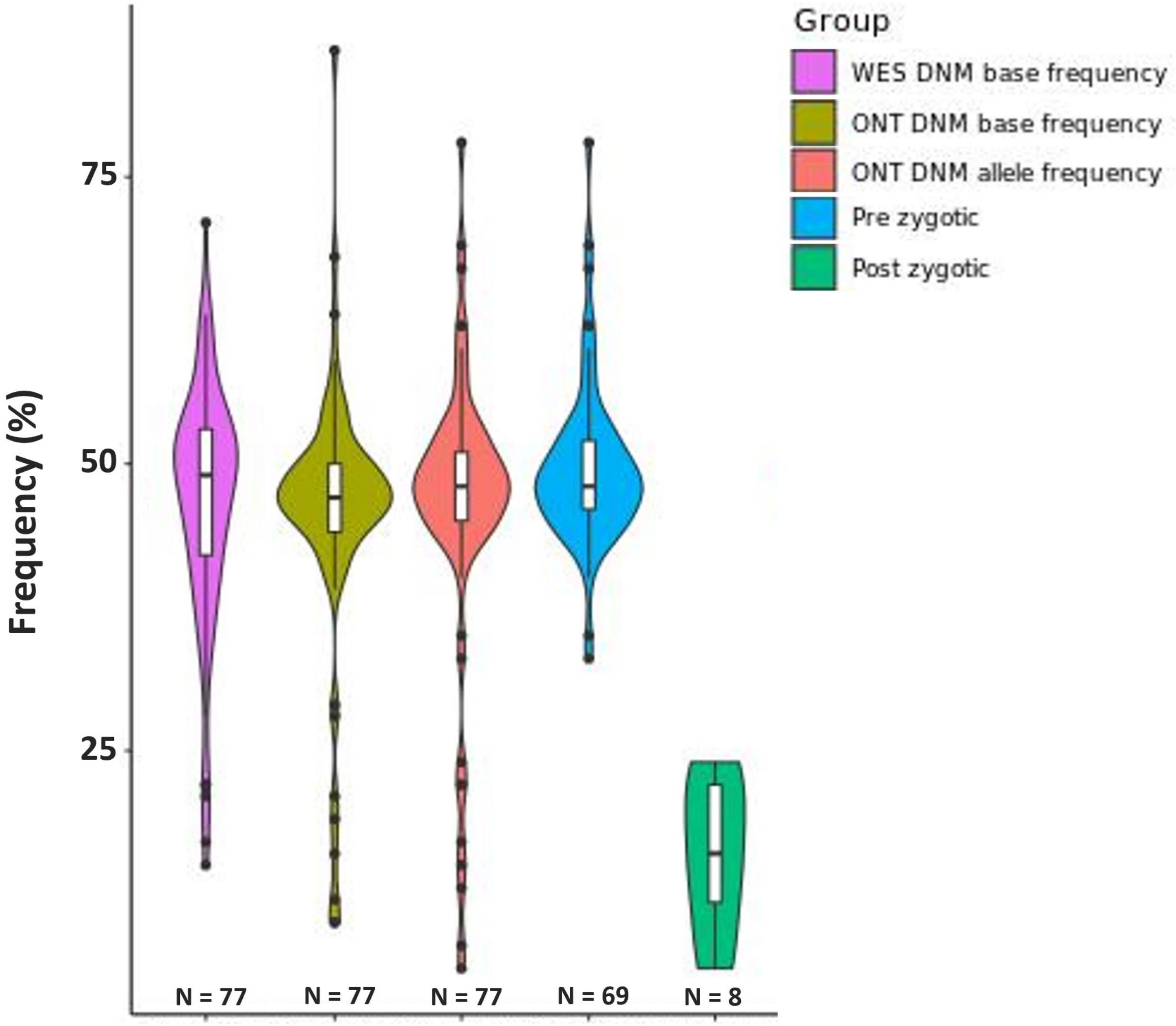

Supplementary Figure 5 Violin plot comparing the WES DNM nucleotide base frequencies, ONT DNM nucleotide base frequencies, ONT DNM allele frequencies, ONT DNM prezygotic and postzygotic allele frequencies.

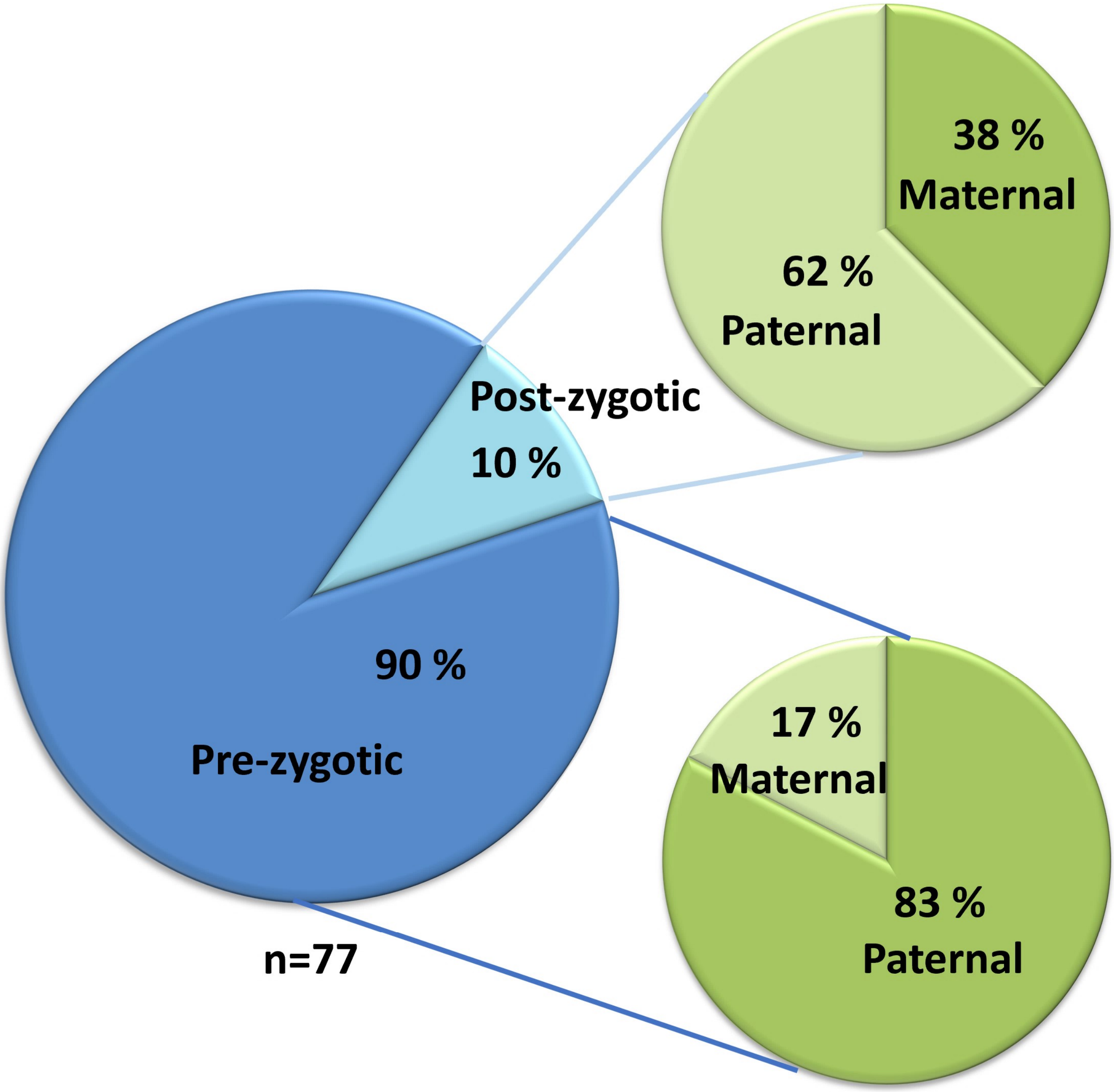

Supplementary Figure 6 Pre and post zygotic illustrative breakdown of the 77 DNMs that could be phased, including the parental origin of the DNMs.

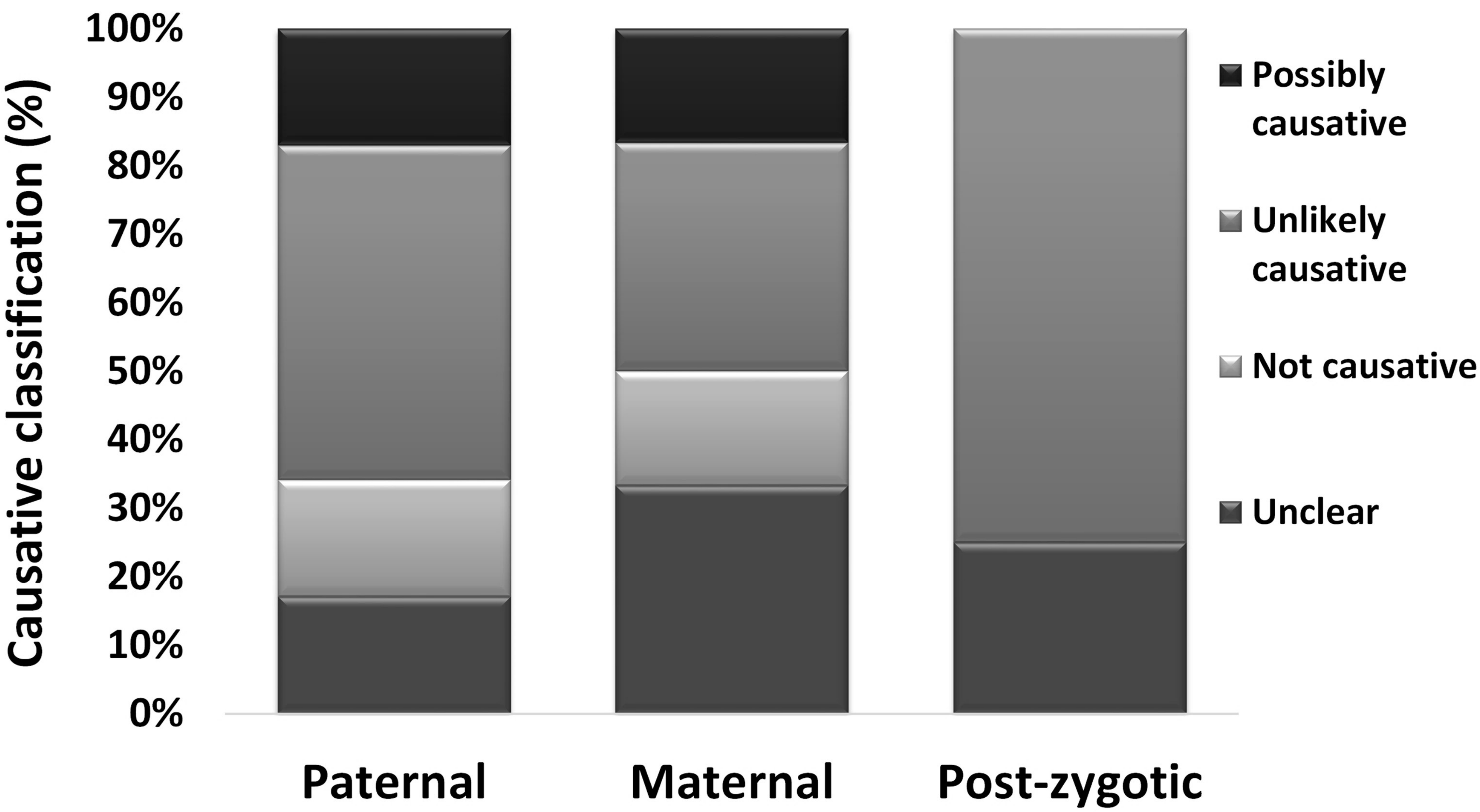

Supplementary Figure 7 Stacked percentage plot looking at parent-of-origin in relation to likelihood of causality.
