# Supplementary material for: Phasing of de novo mutations using a scaled‐up multiple amplicon long‐read sequencing approach
Source: Hum Mutat. 2022 Sep 14;43(11):1545–56. doi: 10.1002/humu.24450 (PMC9826063; doi:10.1002/humu.24450)
Supplement: Supplementary file 3 — Supplementary information. [file HUMU-43-1545-s003.docx]

Supplementary Tables

**Supplementary Table 1** DNM target region primer list.

**Supplementary Table 2** Long-range PCR reaction mixes and running conditions.

**Supplementary Table 3** Base loss breakdown of the 11 barcoded samples after demultiplexing and barcode removal.

**Supplementary Table 4** Point mutation breakdown of different filtering steps.

**Supplementary Table 5** Indel breakdown of different filtering steps.

**Supplementary Table 6** Selected iSNP information for each target.

**Supplementary Table 7** Data analysis for determining the post-zygotic DNMs.

**Supplementary Table 8** DNM target region breakdown of data error and quality.

**Supplementary Table 9** Data overview. Summary tables of supplementary data.

**Supplementary Table 10** Dataset from Smits et al., 2022 used to calculate the percentage of DNMs that have an iSNP within 5kb ranges.

**Supplementary Table 11** Failed target sequenced samples.

**Supplementary Table 12** Phasing and parent-of-origin determination in WES and ONT datasets.

**Supplementary Table 13** Mutant base and allele frequencies with associated pre/post zygotic classifications.

**Supplementary Table 14** DNMs in this dataset with previous causative classifications for male infertility (Oud et al., 2022).

Supplementary Figures

**Supplementary Figure 1 Basic overview of the long-read targeted phasing approach**. Red boxes highlighting failed sample outcomes.

**Supplementary Figure 2 Long range PCR performance and optimisation.** **(a)** change standard to rapid. Percentage comparison of first-time primer success using our standardised approach and primer success after optimisations at increasing target sizes (kb). **(b)** Table of optimisation categories, displaying total target amplification success rates at each stage.

**Supplementary Figure 3 a)** Log10 scatter plot of target amplification sizes in relation to the percentage of false allele coverage of each target and the percentage of false iSNP/DNM base coverage of each target. The trend of the data is highlighted by the logarithmic line of best fit. **b)** Log10 scatter plot of target amplification sizes in relation to the percentage of bases with quality score <5 (see method section ‘Bioinformatics’). The trend of the data is highlighted by the logarithmic line of best fit.

**Supplementary Figure 4 IGV illustration of a DNM showing a comparison of WES and ONT data.** Using long-read targeted sequencing, the ~1700 bp region that these variants span was sequenced and visualized in IGV. Approximately 15000 reads are phased in the ONT data, and the quality and coverage differences between those reads and the WES reads can be observed.

**Supplementary Figure 5** Violin plot comparing the WES DNM nucleotide base frequencies, ONT DNM nucleotide base frequencies, ONT DNM allele frequencies, ONT DNM prezygotic and postzygotic allele frequencies.

**Supplementary Figure 6** Pre and post zygotic illustrative breakdown of the 77 DNMs that could be phased, including the parental origin of the DNMs.

**Supplementary Figure 7** Stacked percentage plot looking at parent-of-origin in relation to likelihood of causality.
